# The differential effects of psychotherapeutic and pharmacologic intervention on symptom severity and functioning in children and adolescents with internalizing problems: a systematic review protocol

**DOI:** 10.1186/s13643-026-03231-9

**Published:** 2026-06-04

**Authors:** Dylan Johnson, Peter Szatmari, Brendan F. Andrade, Karolin Krause, Emma Nolan, Carly Albaum

**Affiliations:** 1 Margaret and Wallace McCain Centre for Child, Youth and Family Mental Health at the Centre for Addiction and Mental Health, Toronto, ON Canada; 2 Department of Applied Psychology and Human Development, University of Toronto, Toronto, ON Canada; 3 Cundill Centre for Child and Youth Depression, Centre for Addiction and Mental Health, Toronto, ON Canada; 4 Department of Psychiatry, University of Toronto, Toronto, ON Canada; 5 Department of Psychiatry, The Hospital for Sick Children, Toronto, ON Canada; 6 Campbell Family Mental Health Research Institute, Centre for Addiction and Mental Health, Toronto, ON Canada; 7 Centre for Research in Epidemiology and Statistics (CRESS), Université Paris Cité and Université Sorbonne Paris Nord, Inserm, INRAe, Paris, France; 8 Department of Psychiatry and Behavioural Neurosciences, McMaster University, Hamilton, ON Canada; 9 Department of Psychology, Queen’s University Belfast, Belfast, Northern Ireland

**Keywords:** Child and Youth Mental Health, Psychotherapy, Pharmacotherapy, Internalizing, Depression, Anxiety, Functioning, Functional impairment, Systematic review, Meta-analysis

## Abstract

**Background:**

Internalizing problems, such as depression and anxiety, are common among children and adolescents, contributing significantly to impairment in various domains of functioning, including academic, social, and family life. While pharmacologic and psychotherapeutic interventions are effective in reducing symptoms, it remains unclear whether these treatments have similar effects to improve functioning. Functional impairment in youth can have long-term consequences, including increased risks for mental health issues, physical health problems, and social difficulties. Despite its importance, functional improvement is often overlooked in mental health intervention research. This study aims to systematically review and meta-analyze interventions that target internalizing symptoms, and assess both symptom reduction and improvements in functioning as treatment outcomes.

**Methods:**

This systematic review and meta-analysis will follow the Preferred Reporting Items for Systematic Review and Meta-Analysis guidelines. A comprehensive search of electronic databases, including CINAHL, EMBASE, MEDLINE, and PsycINFO, will identify randomized controlled trials published between 2014 and 2024 that evaluate psychotherapeutic or pharmacologic treatments for depressive or anxiety symptoms in children and adolescents. Eligible studies will measure both symptom severity and functioning outcomes. Data will be extracted independently by two reviewers, and effect sizes will be calculated using standardized mean differences. Multilevel meta-analytic procedures will be employed to assess both mean differences and variability within studies. Moderator analyses will explore factors such as treatment type, internalizing disorder subtype, and sociodemographic factors. Risk of bias will be assessed using the Cochrane Risk of Bias 2 tool, and the Grading of Recommendations, Assessment, Development, and Evaluations framework will guide the synthesis of the evidence.

**Discussion:**

This review aims to provide a comprehensive understanding of the effectiveness of mental health interventions for youth with internalizing disorders, focusing on both reductions in symptom severity and improvements in functioning. The findings will contribute to the development of clinical guidelines and treatment recommendations, emphasizing the importance of addressing functional outcomes in addition to symptom reduction. The results will have implications for the design and evaluation of future interventions aimed at improving the well-being of children and adolescents.

**Systematic review registration:**

This systematic review is registered in the PROSPERO database (CRD42024610658).

**Supplementary Information:**

The online version contains supplementary material available at 10.1186/s13643-026-03231-9.

## Introduction

### Rationale

Internalizing problems, such as depression and anxiety, are highly prevalent in pediatric populations and significantly contribute to difficulties in functioning across multiple domains (e.g., adaptive, interpersonal, academic) and settings (e.g., home, school, community) [1–3]. In children and youth, functioning refers to the capacity to adapt to the demands of home, school, and community life [4, 5], and emphasizes the ability to meet age- and culturally specific expectations in different domains, including academic achievement, peer relationships, leisure, family functioning, and adaptive skills (e.g., communication, health and safety, self-care). Within each domain, functioning exists along a continuum, where competency or capacity are represented at one end, and impairment at the other [5]. For internalizing problems (i.e., depression, anxiety), symptoms and functioning are closely related. Symptoms, such as anhedonia, low mood, or excessive worrying, can negatively impact a child’s capacity to adapt to their demands, which may manifest as poor academic performance, interpersonal conflict, or reduced engagement in leisure activities. Functional impairment associated with mental health challenges is a key diagnostic criterion (i.e., DSM-5-TR; ICD-11) for internalizing disorders, and carries long-term risks that can persist into adulthood, including further mental health complications, physical health challenges, social drift, delinquency, homelessness, and suicide [6–9]. Youth with more severe functional impairments are also more likely to access outpatient mental health services as compared to those with less severe or no functioning difficulties [10].

In the context of mental health research with children and adolescents, functioning is often not examined as a primary outcome [11]. For example, a systematic review of outcomes measured in adolescent depression studies indicated 94% of studies measured symptom severity while only 52% measured functioning [12]. There is often an assumption that symptom reduction will directly lead to improved functioning, yet the magnitude of association between the symptoms and functioning is only moderate [2, 13], suggesting functioning can fluctuate independent from symptom severity, and vice versa. Prioritizing symptom reduction over functional improvement as the primary outcome in the treatment of pediatric depression and anxiety can obscure the true impact of interventions, with potential consequences for meeting patient needs and effectively allocating resources [14, 15]. Further, stakeholder consensus projects, including those conducted by the International Consortium for Health Outcomes Measurement (ICHOM), have shown that adolescents place high importance on functional improvements [12, 16], and treatments that target improvements in daily functioning over symptom alleviation are more appealing to adolescents [17], underscoring the need for treatments that address more than just symptom severity. While pharmacologic and psychotherapeutic interventions can be effective in reducing symptom severity [18, 19], it is less clear whether treatment may also support improvements in functioning. Determining treatment efficacy based solely on symptom reduction potentially limits the acceptability and relevance of mental health interventions that are evaluated for use with youth. To fully understand treatment effectiveness, research evaluating mental health interventions for children and youth should assess both internalizing symptom severity and functioning.

Based on this gap, the following protocol proposes a systematic review and meta-analysis aimed at addressing the following question: In children and adolescents with depressive or anxious symptoms, how do psychotherapeutic or pharmacologic interventions, compared to any control group, affect symptom severity and functioning? The protocol follows the Preferred Reporting Items for Systematic Review and Meta-Analysis Protocols (PRISMA-P) guidelines (supplementary material; Table S1). While similar reviews have been conducted, these studies have primarily focused on non-pediatric samples [14, 20–27], lacked quantitative meta-analyses [14, 20, 28], or did not evaluate both functioning and symptoms [20, 21, 24, 29, 30]. In addition, while traditional meta-analyses primarily focus on comparing mean differences between groups, they often overlook the variability within outcomes [31]. This is a critical limitation because the distribution of treatment effects can reveal important insights that mean differences alone cannot provide. Thus, this study will add to existing literature by updating prior reviews, while addressing this gap by conducting a systematic review and meta-analysis of pediatric samples with internalizing symptoms, in which both symptom severity and functioning were measured.

### Objectives

1. Systematically review and catalogue the existing literature on pediatric psychotherapeutic and/or pharmacologic interventions for internalizing symptoms and which include outcomes of both symptom severity and functioning.
2. Conduct a meta-analysis that compares mean and dispersion differences between symptom severity and functioning across eligible randomized controlled trials, utilizing standardized mean differences and measures of variability.
3. Investigate effect differences across important moderating factors including treatment type, internalizing type (i.e., anxiety v. depression), and other important clinical and sociodemographic factors.
4. Assess the risk of bias across all included articles including selection bias, performance bias, detection bias, attrition bias, and reporting bias, as well as approaches to multiplicity of outcomes.
5. Apply the Grading of Recommendations, Assessment, Development, and Evaluations (GRADE) framework to synthesize the cumulative evidence regarding the effectiveness of interventions and provide a summary of clinical practice recommendations based on findings and interpretations.

## Methods

The systematic review and meta-analytic process proposed in this protocol will be conducted in accordance with the Preferred Reporting Items for Systematic Review and Meta-Analysis Protocols (PRISMA) guidelines. A completed PRISMA 2020 Checklist (supplementary material; Table S2) will be included in the supplement of the final draft [32].

### Eligibility criteria

Study PICO-T (Population, Intervention, Comparator, Outcome, Time) is presented in Table 1. Eligible studies will focus on children and adolescents under 2–19 years old (at start of treatment) who have been referred/recommended for treatment of depressive or anxious symptoms, at both clinical and subclinical severity levels, in both clinical and community settings. Where the sample age range exceeds 18 years, articles will be eligible if the mean age of the sample is less than 19 years, or where results can be stratified into eligible age bands. Sensitivity analyses comparing studies included on this basis will be conducted. The review will include any psychotherapeutic (Table 2) or pharmacologic (Table 3) interventions permitting they are targeting depression or anxiety. There will be no restrictions on the types of comparators used (e.g., waitlist, treatment as usual, alternative therapy), provided that a comparator is present. To improve comparability of results and reduce heterogeneity in included studies, outcomes of both symptom severity (depression or anxiety) and functioning (global or domain-specific) must be assessed to be eligible for inclusion, regardless of study prioritization (i.e., primary versus secondary outcomes). As global quality of life has been shown to be an empirically distinct construct relative to functioning, it will not be included as an outcome [33]. However, articles will be included when measures of quality of life include relevant domain-specific subscales of functioning (e.g., social functioning subscale on the PedsQL 4.0) [34]. No limitations will be imposed on the duration of treatment or follow-up, nor on the timing of outcome assessments. Only randomized controlled trials (RCTs) published in English, between 2014 and 2024, and appearing in peer-reviewed journals will be included to ensure relevance to contemporary diagnostic frameworks, intervention approaches, and outcome measurement practices in child and adolescent mental health research. Both feasibility and pilot RCTs will be eligible, although priority will be given to final trial reports when available.

**Table 1:** PICO-T criteria for systematic review

(P)opulation	Children and adolescents aged 3 to 18 years (at the start of treatment) referred for treatment of depressive or anxious symptoms. Studies involving participants aged 19 years or older are eligible if the mean age is less than 19 or if results are stratified by age
(I)ntervention	Any psychotherapeutic or pharmacologic interventions for depression or anxiety
(C)omparator	Any comparator present in the studies, such as waitlist control, usual care, or other active interventions. There is no restriction on the type of comparator as long as one is used
(O)utcome	Symptom severity (depression or anxiety) and functioning (global or domain-specific). Both outcomes must be reported for all included studies
(T)ime	There are no specific limitations on the duration of treatment or follow-up and outcome assessments can occur at any time during the treatment or follow-up period

**Table 2:** Psychotherapeutic treatment types

Acceptance and commitment therapy (ACT)
Cognitive-behavioral therapy (CBT)
Dialectical behavior therapy (DBT)
Emotion-focused therapy (EFT)
Exposure therapy
Family therapy
Group therapy
Interpersonal therapy (IPT)
Mindfulness-based therapy
Narrative therapy
Parent–child interaction therapy (PCIT)
Play therapy
Psychodynamic therapy
Solution-focused brief therapy (SFBT)
Trauma-focused cognitive behavioral therapy (TF-CBT)

List is not exhaustive and will be developed iteratively

**Table 3:** Psychotropic treatment classes

Atypical antidepressants
Benzodiazepines
Monoamine oxidase inhibitors (MAOIs)
Selective serotonin reuptake inhibitors (SSRIs)
Serotonin and norepinephrine reuptake inhibitors (SNRIs)
Tricyclic antidepressants

List is not exhaustive and will be developed iteratively

### Information sources and search strategy

Articles will be identified using electronic bibliographic database searches of *Cumulative Index to Nursing and Allied Health Literature* (CINAHL), *Excerpta Medica dataBASE* (EMBASE), *Medical Literature Analysis and Retrieval System Online* (MEDLINE), and *PsycINFO*. The search strategy will be developed in collaboration with a team of clinicians and a research librarian, as well as similar peer-reviewed systematic reviews [30]. The development of the search strategy will be guided by an analysis of the MeSH (Medical Subject Headings) terms and text words found in the titles, abstracts, and keyword sections of a selection of relevant articles, identified through informal literature searches. Four components of the research question will be incorporated into the search strategy to capture the following: Age of study population (e.g., “child”; “adolescent”), internalizing (e.g., “anxiety”; “depression”), functioning (e.g., “family functioning”; “functional impairment”; “school attendance”), and study design (e.g., “RCT”; “control group”). The search strategy is presented in Table S3. Gray literature sources, such as pre-prints, dissertations, theses, conference proceedings, abstracts, and government or industry reports, will be excluded. The decision to exclude gray literature sources was made to focus on high-quality, peer-reviewed research, which has undergone rigorous scrutiny before publication. If over one year has elapsed between the initial search strategy date (November 21, 2024; see below) and completion of the final manuscript, the search strategy and screening process will be updated to reflect new literature.

### Study records

#### Data management

Abstracts and titles will be exported from databases in Research Information Systems (RIS) formats and imported into Covidence software [35]. All study records will be managed using Covidence to facilitate the identification of duplicate records, organization of references, and screening of studies. Covidence will be used for data extraction, where an extraction sheet will instruct the contents of the extraction process. Records will be regularly backed up and tracked throughout the review process to maintain data integrity.

#### Selection process

The selection process will involve a two-step screening: First, titles and abstracts of identified studies will be screened for eligibility based on pre-defined inclusion/exclusion criteria. Full texts of potentially eligible studies will then be reviewed to confirm eligibility. Both phases of screening (i.e., abstract and full-text) will utilize a blinded, two-reviewer process, whereby study extractors will independently screen abstracts and select a response (i.e., include or exclude). Written training documentation and a team training seminar will be conducted at each stage. Discrepancies will be resolved through consensus or consultation with a senior author. To establish a baseline standard of reliability, a subset of *n* = 30 abstracts will be assigned to each reviewer independently in order to calculate levels of agreement. Should the level of agreement not exceed the required threshold (Cohen’s Kappa ≥ 0.75), eligibility criteria will be reviewed as a team and the process will be repeated until the required threshold is met.

#### Data collection process

Data will be extracted independently by two reviewers using a standardized data extraction form. Extracted data will include study characteristics, participant demographics, intervention details, outcome measures related to symptom severity and functioning and estimates of effect size and dispersion. Data will be consolidated into a final study dataset and any inconsistencies will be discussed and resolved prior to data synthesis.

### Data items

Data items for extraction are detailed in Table 4. This will include characteristics of the article (e.g., author, journal), trial information (e.g., trial period, randomization method, pilot v. pivotal), sample (e.g., treatment arm sizes, sociodemographics), measurement characteristics (e.g., tools for measuring functioning and symptoms, informant type), and analysis characteristics/quantitative information (e.g., central tendency, dispersion measures, main findings, covariates).

**Table 4:** Information fields for extraction from each included article

Category	Information extraction field
Article Characteristics	• First author • Year of publication • Journal name • Country/region of study
RCT Trial Information	• Trial name • Trial period • Trial location • Pivotal v. pilot • Randomization method • Blinding/masking • Allocation concealment • Primary outcomes • Secondary outcomes • Treatment type • Control type
Sample	• Overall sample size • Age • Sex • Race/ethnicity • High-income region v. low- and middle-income region • Comorbidities • Medication • Sample size in treatment arm (used in analysis) • Sample size in control arm (used in analysis) • Dropout rate
Measurement	• Presenting symptom/diagnoses type • Symptom/diagnosis assessment method • Measure(s) of mental health symptoms at baseline/follow-up • Measure(s) of functioning at baseline/follow-up • Primary outcome(s) • Secondary outcome(s) • Covariate adjustment • Informant type
Analysis	• Central tendency measurement type • Dispersion measurement type • Effect size measurement type • Between group(s) central tendency value(s) • Between group(s) dispersion value(s) • Within group(s) central tendency value(s) • Within group(s) dispersion value(s) • Effect size value • Correlation measure between symptoms and functioning • *p*-value(s) for symptoms and functioning effects • Main findings

### Outcomes and prioritization

In determining outcome priority, the primary outcomes for this study will be post-treatment changes in both internalizing symptoms (i.e., anxiety or depression) and functioning, as well as a comparison of treatment effects *between* changes in symptoms and changes in functioning. The secondary outcomes for this study will be subgroup analyses into specific subtypes of internalizing symptoms (e.g., anxiety vs. depression broadly; subtypes including generalized anxiety, social phobia), functioning (e.g., academic, social, global), and treatment modality (e.g., psychotherapeutic v. pharmacologic; subtypes of psychotherapy or medication), where possible. If adequately reported, moderator analyses will be conducted on sex, gender, age, ethnicity, and comorbidity status. Aggregated correlations between functioning and symptom severity will also be calculated, as well as descriptive reporting on the difference in significance (*p* < 0.05) versus non-significance (*p* ≥ 0.05) of both symptom and functioning outcomes. Finally, within-group changes over time will also be investigated in secondary analyses (i.e., pre-post changes in symptoms and functioning within treatment condition).

### Risk of bias in individual studies

The Cochrane Risk of Bias 2 (RoB 2) tool will be adapted to assess for risk of bias within included articles [36]. This measure assesses bias arising from randomization, deviations from intended intervention/protocol, missing outcome data/attrition, measurement, and reporting. This review will involve a thorough evaluation of each domain to ensure a comprehensive understanding of potential biases that may impact the validity of each study and the impact on overall interpretation of synthesized findings. As the review focuses on outcomes for two separate constructs (i.e., symptoms and functioning) and studies will likely include multiple comparisons, the RoB 2 will be supplemented with an assessment of (1) pre-specification of primary and secondary outcomes; and (2) correction for multiple testing (e.g., Bonferroni adjustment, Holm’s step-down procedure).

### Data synthesis

The data synthesis for this proposed project will be a meta-analysis involving the aggregation of effect sizes derived from eligible RCTs investigating the effects of interventions on symptom severity and functioning. Traditionally, meta-analyses focus on comparing mean differences between groups for specified outcomes. However, to enhance our understanding of treatment effects, meta-analyses comparing mean differences between groups for specified outcomes, as well as assessing the variability of the data will be conducted. Standardized mean differences (SMD) will be calculated using Hedges' g to facilitate comparison across studies, allowing for a pooled effect size representation of symptom severity and functioning. In addition, the variance ratio (VR) and coefficient of variation ratio (CVR) will be employed to evaluate group differences in variability [31]. These measures will provide insight into how the spread of data impacts overall effect sizes and the frequency of extreme observations within studies.

Multilevel meta-analytic procedures will be utilized to statistically account for the inherent multilevel structure of the data [37, 38]. This includes participants (level 1) nested within studies (level 2), as well as between-cluster heterogeneity (level 3). As multiple effects will be extracted from the same study (i.e., at minimum, one symptom outcome and one functioning outcome), this method enhances the ability to accurately assess effect sizes and heterogeneity across the studies included in the review, accounting for within and between variability.

Using a random-effects model, pooled effect sizes will be reported separately for both outcomes, alongside assessments of variability through VR and CVR, and will be interpreted according to proposed benchmarks for small (~ 0.2), medium (~ 0.5), and large (~ 0.8) effects. Heterogeneity will be assessed using I2 statistics and explored via moderator analyses, such as comparisons between pilot versus pivotal RCT designs, global versus domain-specific functioning, and psychotherapy versus pharmacotherapy. Publication bias will be evaluated through funnel plots and Egger’s test. Moderator analyses will be explored and developed iteratively based on availability of data, and tested using meta-regression analyses. Analyses will be conducted using the “metafor” package in R software.

If a quantitative meta-analysis is not feasible, an alternative approach will involve conducting a systematic review of studies, narratively synthesizing key themes and patterns related to treatment effects on symptom severity and functioning, and exploring the underlying factors that influence these outcomes.

### Meta-bias and confidence in cumulative evidence

The Grading of Recommendations, Assessment, Development, and Evaluations (GRADE) framework will be used to summarize and present cumulative evidence from this systematic review, as well as guide the making of clinical practice recommendations [39, 40]. GRADE guides the development of a certainty in evidence index, ranging from “very low” (i.e., true effect markedly different from observed effect) to “high” (i.e., confidence that the true effect is similar to the observed effect). The overall confidence in cumulative evidence will be informed by risk of bias, imprecision, inconsistency, indirectness, publication bias, magnitude of effect(s), dose–response gradient, and residual confounding.

## Discussion

This systematic review and meta-analysis will offer a critical contribution to the understanding of how pharmacologic and psychotherapeutic interventions for internalizing symptoms in children and adolescents influence both symptom severity and functioning. While prior research has focused predominantly on symptom alleviation, the current review recognizes that mental health symptoms and functional outcomes are related but distinct constructs. By systematically examining the magnitude and variability of change in both symptom severity and functioning, this study will help clarify whether and to what extent improvements in symptoms translate into meaningful gains in adaptive, academic, and social functioning.

One of the most salient contributions of this review is its focus on functioning as a co-primary outcome, a perspective increasingly prioritized by youth, families, and clinical stakeholders. Adolescents often identify functioning—such as doing well in school, maintaining relationships, and engaging in daily life—as more important to them than simply feeling less anxious or depressed. Yet despite this, many randomized controlled trials continue to neglect functional outcomes or treat them as secondary and exploratory. By including only those studies that measure both outcomes, and by comparing effect sizes and their variability, this review seeks to redress this imbalance and provide a more holistic assessment of treatment impact.

This review employs robust methodological approaches—including dispersion metrics and multilevel modeling—to evaluate not only average treatment effectiveness but also variability in response, which is especially important in pediatric populations where developmental and contextual factors influence outcomes. By examining moderators such as treatment type, symptom subtype, and demographic or clinical variables, the review identifies subgroups of youth who may benefit most from specific interventions, advancing a more precision-oriented approach to care. Applying the GRADE framework to assess evidence quality, the review aims to inform clinical guidelines and emphasizes the importance of functional outcomes in both research and practice. Ultimately, this work supports a developmentally grounded, person-centered model of pediatric mental health care that prioritizes young people’s ability to thrive in everyday contexts.

## Supplementary Information

Supplementary Material 1. Table S1: PRISMA-P 2015 Checklist.

Supplementary Material 2. Table S2: PRISMA 2020 Checklist.

Supplementary Material 3. Table S3: Search Strategy.

## Data Availability

All data extracted and analyzed in this systematic review and meta-analysis will be made available at the time of publication of the final manuscript.
