# Supplementary material for: The differential effects of psychotherapeutic and pharmacologic intervention on symptom severity and functioning in children and adolescents with internalizing problems: a systematic review protocol
Source: Syst Rev. 2026 Jun 4;15:214. doi: 10.1186/s13643-026-03231-9 (PMC13449698; doi:10.1186/s13643-026-03231-9)
Supplement: Supplementary file 3 [file 13643_2026_3231_MOESM3_ESM.docx]

| Table S3: Search Strategy | |
| --- | --- |
| **EMBASE** | |
| 1 | Child/ or Adolescent/ or (child* or adole* or boy or girl or teen* or Youth? or "Young people" or "Young person?" or juvenile* or highschool* or "high-school*" or "school age*" or p?ediatric*).mp. |
| 2 | social anxiety/ or anxiety/ or separation anxiety/ or anxiety disorder/ or generalized anxiety disorder/ or anxiety neurosis/ or performance anxiety/ or (anxi* or selective mutis* or phobia* or panic* or agoraphobi* or GAD or OCD or obsessi* or compul*).mp. or minor depression/ or chronic depression/ or "mixed anxiety and depression"/ or atypical depression/ or adolescent depression/ or major depression/ or long term depression/ or depression/ or depressive disorder/ or depressive disorder, major/ or depress*.mp. |
| 3 | (abilit* or perform*)) or (scholastic adj2 (achievement or accomplish*)) or gradepoint average or homework or grade) or ((School* adj2 (attend* or termination)) or dropout* or absent* or days miss*) or ((Famil* adj3 (function* or relation* or problem* or dynamic* or life* or environment*)) or (relations adj2 (sibling* or parent-child or parent child or parent* or mother-child)) or (satisfaction* adj2 famil*)) or ((daily adj2 (activit* or routine* or living* or life*)) or (activit* adj2 (daily or recreation* or extracurricular)) or after school or after-school or activities of daily living or (sport* adj2 (participat* or engage* or attend*)) or hobb*) or ((((function* adj2 (adaptive* or impair*)) or (impair* adj2 life*) or life interference or quality of life or real life outcome* or secondary outcome* or life impact or Strengths) and Difficulties questionnaire) or Child Behaviour Checklist or Childrens Global Assessment Scale or Global Assessment of Functioning or impair* or global functioning or Columbia impairment scale or Children's Global Assessment Scale or Adolescent Life Interference Scale or Sheehan Disability Scale or QoL or HRQOL or PedsQL or QoL-ChYMH or PQ-LES-Q)).mp. |
| 4 | (controlled clinical trial or RCT or randomi#ed controlled trial or randomi#ed or randomi#ation or randomly or (random* adj3 (allocate* or assign* or control* or treat*)) or (control adj3 (trial* or study or studies)) or ((single* or doubl* or tripl*) adj3 (blind* or mask*)) or clinical trial or (quasi adj2 (experimental or random*)) or (group adj2 (control or treatment))).mp. |
| 5 | 1 AND 2 AND 3 AND 4 |
| 6 | limit 5 to (english language and yr="2014 -Current") |
| **MEDLINE** |  |
| 1 | Child/ or Adolescent/ or (child* or boy or girl or teen* or Youth? or "Young people" or "Young person?" or juvenile* or highschool* or high-school* or "school age*" or p?ediatric*).mp. |
| 2 | Anxiety Disorders/ or Anxiety/ or Anxiety, Separation/ or Performance Anxiety/ or Depression/ or (anxi* or selective mutis* or phobia* or panic* or agoraphobi* or GAD or depress* or OCD or obsessi* or compul*).mp. |
| 3 | (socioemotional functioning or socio-emotional functioning or peer relations or friendship or peer interaction* or friends* or popular* or bully* or peer reject* or (social* adj2 (function* or skill* or competenc* or adjust* or interact* or difficult* or problem* or issue* or adaptive behavio#r* or abilit*)) or (interpersonal adj2 (competenc* or skill*)) or ((school adj2 (perform* or achiev* or work* or engage*)) or (academic* adj2 (achieve* or perform* or engage*)) or (education* adj2 attain*) or (cognit* adj2 (abilit* or perform*)) or (scholastic adj2 (achievement or accomplish*)) or gradepoint average or homework or grade) or ((School* adj2 (attend* or termination)) or dropout* or absent* or days miss*) or ((Famil* adj3 (function* or relation* or problem* or dynamic* or life* or environment*)) or (relations adj2 (sibling* or parent-child or parent child or parent* or mother-child)) or (satisfaction* adj2 famil*)) or ((daily adj2 (activit* or routine* or living* or life*)) or (activit* adj2 (daily or recreation* or extracurricular)) or after school or after-school or activities of daily living or (sport* adj2 (participat* or engage* or attend*)) or hobb*) or ((((function* adj2 (adaptive* or impair*)) or (impair* adj2 life*) or life interference or quality of life or real life outcome* or secondary outcome* or life impact or Strengths) and Difficulties questionnaire) or Child Behaviour Checklist or Childrens Global Assessment Scale or Global Assessment of Functioning or impair* or global functioning or Columbia impairment scale or Children's Global Assessment Scale or Adolescent Life Interference Scale or Sheehan Disability Scale or QoL or HRQOL or PedsQL or QoL-ChYMH or PQ-LES-Q)).mp. |
| 4 | RCT.mp. or controlled clinical trial.ti,ab. or randomi#ed controlled trial.ti,pt. or randomi#ed.mp. or randomi#ation.ti,ab. or randomly.ab. or (random* adj3 (allocate* or assign* or control* or treat*)).ti,ab. or (control adj3 (trial* or study or studies)).ti,ab. or ((single* or doubl* or tripl*) adj3 (blind* or mask*)).mp. or clinical trial.ti,ab. or (quasi adj2 (experimental or random*)).ti,ab. or (group adj2 (control or treatment)).ab. |
| 5 | 1 AND 2 AND 3 AND 4 |
| 6 | limit 5 to (english language and yr="2014 -Current") |
| **PsycINFO** |  |
| 1 | Pediatrics/ or (adolesc* or child* or boy or girl or teen* or Youth? or "Young people" or "Young person?" or juvenile* or highschool* or "high-school*" or "school age*" or p?ediatric*).mp. |
| 2 | Anxiety Disorders/ or Anxiety/ or Generalized Anxiety Disorder/ or Separation Anxiety/ or Separation Anxiety Disorder/ or Performance Anxiety/ or Social Anxiety/ or Atypical Depression/ or Major Depression/ or Recurrent Depression/ or Treatment Resistant Depression/ or (anxi* or selective mutis* or phobia* or panic* or agoraphobi* or GAD or depress* or OCD or obsessi* or compul*).mp. |
| 3 | (socioemotional functioning or socio-emotional functioning or peer relations or friendship or peer interaction* or friends* or popular* or bully* or peer reject* or (social* adj2 (function* or skill* or competenc* or adjust* or interact* or difficult* or problem* or issue* or adaptive behavio#r* or abilit*)) or (interpersonal adj2 (competenc* or skill*)) or ((school adj2 (perform* or achiev* or work* or engage*)) or (academic* adj2 (achieve* or perform* or engage*)) or (education* adj2 attain*) or (cognit* adj2 (abilit* or perform*)) or (scholastic adj2 (achievement or accomplish*)) or gradepoint average or homework or grade) or ((School* adj2 (attend* or termination)) or dropout* or absent* or days miss*) or ((Famil* adj3 (function* or relation* or problem* or dynamic* or life* or environment*)) or (relations adj2 (sibling* or parent-child or parent child or parent* or mother-child)) or (satisfaction* adj2 famil*)) or ((daily adj2 (activit* or routine* or living* or life*)) or (activit* adj2 (daily or recreation* or extracurricular)) or after school or after-school or activities of daily living or (sport* adj2 (participat* or engage* or attend*)) or hobb*) or ((((function* adj2 (adaptive* or impair*)) or (impair* adj2 life*) or life interference or quality of life or real life outcome* or secondary outcome* or life impact or Strengths) and Difficulties questionnaire) or Child Behaviour Checklist or Childrens Global Assessment Scale or Global Assessment of Functioning or impair* or global functioning or Columbia impairment scale or Children's Global Assessment Scale or Adolescent Life Interference Scale or Sheehan Disability Scale or QoL or HRQOL or PedsQL or QoL-ChYMH or PQ-LES-Q)).mp. |
| 4 | controlled clinical trial.ti,ab. or RCT.mp. or randomi#ed controlled trial.ti,pt. or randomi#ed.mp. or randomi#ation.ti,ab. or randomly.ab. or (random* adj3 (allocate* or assign* or control* or treat*)).ti,ab. or (control adj3 (trial* or study or studies)).ti,ab. or ((single* or doubl* or tripl*) adj3 (blind* or mask*)).mp. or clinical trial.ti,ab. or (quasi adj2 (experimental or random*)).ti,ab. or (group adj2 (control or treatment)).ab. |
| 5 | 1 AND 2 AND 3 AND 4 |
| 6 | limit 5 to (english language and yr="2014 -Current") |
| **CINAHL** |  |
| S1 | (MH "Anxiety") OR (MH "Anxiety Disorders") OR (MH "Separation Anxiety") OR (MH "Social Anxiety Disorders") OR (MH "Generalized Anxiety Disorder") OR TI anx* OR AB anx* OR (MH "Depression") OR TI depress* OR AB depress* OR TI OCD OR AB OCD OR TI obsessi* OR AB obsessi* OR TI compul* OR AB compul* |
| S2 | (MH "Child") OR (MH "Adolescence") OR TI child* OR AB child* OR TI adole* OR AB adole* OR TI boy OR AB boy OR TI girl OR AB girl OR TI teen* OR AB teen* OR TI youth* OR AB youth* OR TI "young people" OR AB "young people" OR TI "young person" OR AB "young person" OR TI juvenile* OR AB juvenile* OR TI highschool* OR AB highschool* OR TI "high-school*" OR AB "high-school*" OR TI "school age*" OR AB "school age*" OR TI p?ediatric* OR AB p?ediatric* |
| S3 | TI socioemotional functioning OR AB socioemotional functioning OR TI socio-emotional functioning OR AB socio-emotional functioning OR TI peer relations OR AB peer relations OR TI friendship OR AB friendship OR TI peer interaction* OR AB peer interaction* OR TI friends* OR AB friends* OR TI popular* OR AB popular* OR TI bully* OR AB bully* OR TI peer reject* OR AB peer reject* OR TI (social* adj2 (function* OR skill* OR competenc* OR adjust* OR interact* OR difficult* OR problem* OR issue* OR adaptive behavio#r* OR abilit*)) OR AB (social* adj2 (function* OR skill* OR competenc* OR adjust* OR interact* OR difficult* OR problem* OR issue* OR adaptive behavio#r* OR abilit*)) OR TI (interpersonal adj2 (competenc* OR skill*)) OR AB (interpersonal adj2 (competenc* OR skill*)) OR TI ((school adj2 (perform* OR achiev* OR work* OR engage*)) OR AB ((school adj2 (perform* OR achiev* OR work* OR engage*))) OR TI ((academic* adj2 (achieve* OR perform* OR engage*)) OR AB ((academic* adj2 (achieve* OR perform* OR engage*))) OR TI ((education* adj2 attain*) OR AB ((education* adj2 attain*))) OR TI ((cognit* adj2 (abilit* OR perform*)) OR AB ((cognit* adj2 (abilit* OR perform*))) OR TI ((scholastic adj2 (achievement OR accomplish*)) OR AB ((scholastic adj2 (achievement OR accomplish*))) OR TI gradepoint average OR AB gradepoint average OR TI homework OR AB homework OR TI grade OR AB grade OR TI ((School* adj2 (attend* OR termination)) OR AB ((School* adj2 (attend* OR termination))) OR TI dropout* OR AB dropout* OR TI absent* OR AB absent* OR TI days miss* OR AB days miss* OR TI ((Famil* adj3 (function* OR relation* OR problem* OR dynamic* OR life* OR environment*)) OR AB ((Famil* adj3 (function* OR relation* OR problem* OR dynamic* OR life* OR environment*))) OR TI (relations adj2 (sibling* OR parent-child OR parent child OR parent* OR mother-child)) OR AB (relations adj2 (sibling* OR parent-child OR parent child OR parent* OR mother-child)) OR TI (satisfaction* adj2 famil*) OR AB (satisfaction* adj2 famil*) OR TI ((daily adj2 (activit* OR routine* OR living* OR life*)) OR AB ((daily adj2 (activit* OR routine* OR living* OR life*))) OR TI ((activit* adj2 (daily OR recreation* OR extracurricular)) OR AB ((activit* adj2 (daily OR recreation* OR extracurricular))) OR TI after school OR AB after school OR TI after-school OR AB after-school OR TI activities of daily living OR AB activities of daily living OR TI ((sport* adj2 (participat* OR engage* OR attend*)) OR AB ((sport* adj2 (participat* OR engage* OR attend*))) OR TI hobb* OR AB hobb* OR TI ((((function* adj2 (adaptive* OR impair*)) OR AB ((function* adj2 (adaptive* OR impair*))) OR TI (impair* adj2 life*) OR AB (impair* adj2 life*) OR TI life interference OR AB life interference OR TI quality of life OR AB quality of life OR TI real life outcome* OR AB real life outcome* OR TI secondary outcome* OR AB secondary outcome* OR TI life impact OR AB life impact OR TI Strengths) AND Difficulties questionnaire) OR AB (Strengths) AND Difficulties questionnaire) OR TI Child Behaviour Checklist OR AB Child Behaviour Checklist OR TI Childrens Global Assessment Scale OR AB Childrens Global Assessment Scale OR TI Global Assessment of Functioning OR AB Global Assessment of Functioning OR TI impair* OR AB impair* OR TI global functioning OR AB global functioning OR TI Columbia impairment scale OR AB Columbia impairment scale OR TI Children's Global Assessment Scale OR AB Children's Global Assessment Scale OR TI Adolescent Life Interference Scale OR AB Adolescent Life Interference Scale OR TI Sheehan Disability Scale OR AB Sheehan Disability Scale OR TI QoL OR AB QoL OR TI HRQOL OR AB HRQOL OR TI PedsQL OR AB PedsQL OR TI QoL-ChYMH OR AB QoL-ChYMH OR TI PQ-LES-Q OR AB PQ-LES-Q |
| S4 | TI controlled clinical trial OR AB controlled clinical trial OR TI RCT OR AB RCT OR TI randomi#ed controlled trial OR AB randomi#ed controlled trial OR TI randomi#ed OR AB randomi#ed OR TI randomi#ation OR AB randomi#ation OR TI randomly OR AB randomly OR TI (random* adj3 (allocate* OR assign* OR control* OR treat*)) OR AB (random* adj3 (allocate* OR assign* OR control* OR treat*)) OR TI (control adj3 (trial* OR study OR studies)) OR AB (control adj3 (trial* OR study OR studies)) OR TI ((single* OR doubl* OR tripl*) adj3 (blind* OR mask*)) OR AB ((single* OR doubl* OR tripl*) adj3 (blind* OR mask*)) OR TI clinical trial OR AB clinical trial OR TI (quasi adj2 (experimental OR random*)) OR AB (quasi adj2 (experimental OR random*)) OR TI (group adj2 (control OR treatment)) OR AB (group adj2 (control OR treatment)) |
| S5 | S1 AND S2 AND S3 AND S4 |
| S6 | S5: Limiters - Publication Date: 20140101-20250105; English Language |
